# Pharmaceutical Pricing and R&D as a Global Public Good

**DOI:** 10.1002/hec.70105

**Published:** 2026-04-07

**Authors:** H. E. Frech, Mark V. Pauly, William S. Comanor, Joseph R. Martinez

**Affiliations:** ^1^ Department of Economics University of California Santa Barbara California USA; ^2^ Health Care Management Department University of Pennsylvania Philadelphia Pennsylvania USA; ^3^ Wharton School of the University of Pennsylvania Philadelphia Pennsylvania USA; ^4^ Fielding School of Public Health University of California Los Angeles California USA

**Keywords:** free riding, innovation, international cooperation, international pharmaceutical pricing, pharmaceutical research and development as a global public good

## Abstract

This paper examines the international variation in the prices of branded pharmaceuticals. We consider short‐run profits, or quasi‐rents, as representing each country's contribution toward the global public good of therapeutic information embodied in new pharmaceuticals. We characterize globally optimal contributions through the Samuelson‐Lindahl criteria and contrast the resulting outcomes with the Nash noncooperative equilibrium as developed in the Olson‐Zeckhauser theory of international alliances. That theory predicts both the undersupply of public goods and the “exploitation” of large countries by small ones. We calculate national contributions to the global public good with data from a recent RAND report. Other countries' contributions resulting from their prices are much lower than those in the United States, but their free riding is not complete. We also find that country size is a powerful determinant of contributions and that larger countries contribute disproportionately more. Finally, we suggest a cooperative policy approach that would move us closer to optimality with health benefits for all countries.

## 1 Introduction

There is strong evidence that US prices for branded pharmaceuticals are much higher generally than those paid for the same products in the rest of the world (ROW). As shown in the recent RAND Report (Mulcahy et al. 2021, 49), US prices for “Brand Name Originator Drugs” are more than twice the average found in 32 OECD developed countries, even after adjusting US prices for rebates and discounts.

While the RAND Report does not explain these differences, that is our purpose here. We offer two accounts: the first is a normative evaluation of whether current cross‐country variation in the prices of branded pharmaceuticals is globally optimal. And second, we construct a positive behavioral model of what determines existing price patterns. We then propose a policy direction to move outcomes closer to the international optimum.

For these purposes, we employ the economic theory of public goods (Samuelson 1954, 1955). By interpreting the new information discovered thorough industry research and development (R&D) as a public good embodied in branded pharmaceuticals, we derive implications for their international prices and quantities. In this effort, we rely on two strains of the economic literature. In the normative analysis, we apply the classic Samuelson‐Lindahl (SL) approach to describe globally optimal investment in pharmaceutical research and development. In contrast, for the positive analysis, we apply the classic Olson and Zeckhauser (1966) theory of alliances (OZ). We then provide empirical evidence on how close existing patterns of relative prices provided are to the behavioral equilibrium in OZ or the global optimum in SL.

## 2 Pharmaceutical Innovation and Pricing

Since R&D outlays are entirely a feature of the branded industry, this analysis is limited to that segment of pharmaceutical supply.

Discovering new ideas to treat disease, along with establishing the safety and efficacy of products, is an expensive proposition with costs estimated at more than $3 billion per new pharmaceutical product (DiMasiGrabowski et al. 2016). (Other estimates are lower, such as the $1.6 billion in the estimates relied upon by the Congressional Budget Office (U.S. CBO, 2019; Wouters et al. 2020, 850)). The scientific and practical information created by a pharmaceutical company's own R&D efforts, or purchased from independent companies, is incorporated in its medicines offered for sale.

Since the cost of creating scientific and practical knowledge is largely borne before a single unit of the pharmaceutical can be sold, these expenditures represent sunk costs that support all units sold. The basic biomedical and biochemical research that underlies much applied pharmaceutical research is provided as well by national governments. Indeed, the US Congressional Budget Office found that government supported R&D is highly complementary to industry R&D rather than being an alternative (U.S. CBO, 2021, 19). (See also the corresponding study, albeit one dealing with a different global public good (Kyle et al. 2017)). As with sunk costs generally, R&D investments are recovered from differences between the revenues received and payments for production and distribution costs; by the difference between prices and short‐run marginal costs.

Because past R&D costs have already been paid, they do not directly influence the prices charged for already‐discovered pharmaceuticals. Instead, R&D outlays depend on the anticipated incremental profits to be gained from future new pharmaceuticals and the long‐run incremental costs of inventing, testing, obtaining approval and initially promoting new pharmaceuticals. Much of this outlay is not directed at new basic science but instead to clinical trials and other development expenditures. Expected markups of prices over marginal production and distribution costs incentivize the R&D efforts designed to develop advanced pharmaceutical agents.

We suggest that revenues exceeding production and distribution costs for new branded pharmaceuticals should not be viewed as excess returns with no economic function, but instead largely that act to incentivize investment in the knowledge embodied in innovative pharmaceuticals. One can think of them as an “investment in innovation” (Berndt et al. 2011, 251). These revenues also incentivize the promotion and rapid diffusion of innovative pharmaceuticals that expands their returns and contributes to the resulting health benefits. We view these revenues as largely as “quasi‐rents” that support essential sunk costs rather than simple monopoly profits. Indeed, if there were no barriers to innovation and entry, returns on investment in new pharmaceuticals would be expected to earn competitive rates of return. There have been a couple of broad‐based studies directly on this point. Grabowski, Vernon and DiMasi report that the aggregate rate of return from new drugs is close to the cost of capital. They estimated these returns at 11.5% as compared to the estimated cost of capital of 11.0%. (2002). Earlier work showed slightly higher rates of return, especially for the UK (Simmons et al. 1981). This point of view offers a different lens through which to view the bulk of pharmaceutical profits than is commonly used (DeAngelis 2016).

There is considerable empirical support for the connections among pharmaceutical prices, R&D outlays and pharmaceutical innovation. In an early study, Comanor (1965) provided direct evidence of the association between R&D efforts and the number of new pharmaceuticals introduced, where each is weighted by its sales in the first 2 years following introduction. A related result is Scherer's finding of a direct connection between overall profit margins and R&D spending (2001). Civan and Maloney (2009) also find that current pharmaceutical prices are an important determinant of R&D spending. Furthermore, Santerre et al. (2005) report that the prices charged for current pharmaceuticals are an important determinant of the number of prospective pharmaceuticals in the R&D pipeline.

In another important paper, Acemoglu and Linn report that innovation and the entry of new pharmaceuticals is driven by prospective market expenditures (2004. 1051, 1061). In an extensive econometric study, Dubois et al., find a significant elasticity of pharmaceutical innovation with respect to market size in terms of expected revenues or profits. Overall, they conclude that expected worldwide profits of $2.5 billion at initiation of R&D are necessary to incentivize the introduction of a new pharmaceutical (2015, 861, 862). Dugan and Scott‐Morton find that the introduction of Medicare drug coverage (Part D) raised demand and total profits to pharmaceutical firms, at the same time reducing prices (2010).

From these studies, it follows that US policy discussions of pharmaceutical pricing issues commonly include their likely effects on the incentives for innovation (Filson 2012; Goldman and Lakdawalla 2018; Lakdawalla 2018; U.S. CBO 2021). An alternate approach employed by some countries is to adopt “Value Based Pricing” rules that reward sellers of innovative pharmaceuticals. However, beyond such actions, there are few concerns expressed outside of the US about the global supply of innovative pharmaceuticals.

The higher prices charged for pharmaceuticals during their effective patent lives are a direct result of US law. The Hatch‐Waxman Act of 1984 included provisions designed specifically to incentivize pharmaceutical innovation. See the following judicial interpretation of that law:

The Act emerged from Congress' efforts to balance two conflicting policy objectives: to induce name‐brand pharmaceutical firms to make the investments necessary to research and develop new drug products, while simultaneously enabling competitors to bring cheaper, generic copies of those drugs to market.

(*Abbott Labs v. Young*, 190, 192 F2d 984 1990)

In the analysis below, we consider the information generated by R&D as a public good that is embodied in innovative pharmaceuticals and made temporarily excludable through being incorporated in patent‐protected products.

## 3 Pharmaceutical Information as a Global Public Good

Although pharmaceuticals themselves are private goods, the information embodied in them is the essential public good that we consider here. The classic example of a public good is information (Stiglitz 1999; Cruzet et al. 2022, 3). While often costly to produce, the use of new information by one person or entity does not restrict the amounts available for others. Indeed, the presence of “non‐rivalry” is an essential feature of any public good in that its value is not diminished by an additional user but remains fully available for others. Furthermore, information is both readily disseminated and difficult to control.

In principle, a single world body could decide how to regulate patent exclusivity to incentivize the optimal supply of global R&D, considering both the welfare benefits of new knowledge and the welfare loss due to both the higher prices charged for the products supplied and the cost of R&D, marketing/information, production, and physical distribution. However, these problems are far more complicated when there are separate national markets with different national authorities who have little concern for, or even knowledge of, global welfare.

Individual countries design or accept institutional arrangements through which prices are determined. Following a detailed review of international health care systems, Rice aptly points out that “nearly all of the countries either set country‐level pharmaceutical prices or engage in explicit negotiations with manufacturers” through a central authority However, he continues, “none of these activities are carried out by the US federal government.” (Rice 2021, 209). To be sure, US market conditions have changed recently for a small number of high‐volume pharmaceuticals sold to Medicare beneficiaries.

A common perception is that, outside the US, national free riding practices are widespread. See for example the Council of Economic Advisors Report, which states that “foreign governments can insist on a price” just above marginal cost of production (2018. 14) and thereby contribute little to the R&D cost of pharmaceutical innovation. Furthermore, in a recent editorial, Hooper and Henderson assert that the ROW countries free ride by compelling pharmaceutical firms to accept prices just above the marginal cost of production and that firms accept these low prices because “some money is better than no money” (2022). In the analysis to follow, we examine these propositions both theoretically and empirically.

## 4 The Lindahl Normative Model

We start by considering the United States (or any other country) in isolation. To determine the optimal level of the public good represented by the flow of new pharmaceuticals, we employ a demand or marginal benefit function for the population of the country along with a production function for the supply of new pharmaceuticals. For this purpose, assume that each citizen of country *U* places a value *V*_*i*_ on the health improvement anticipated to follow from a new innovative pharmaceutical. The population‐level marginal benefit or demand curve at any quantity or flow of new pharmaceuticals *Q* is then determined by the vertical sum of these marginal valuations over all persons in the country.

**FIGURE 1:**
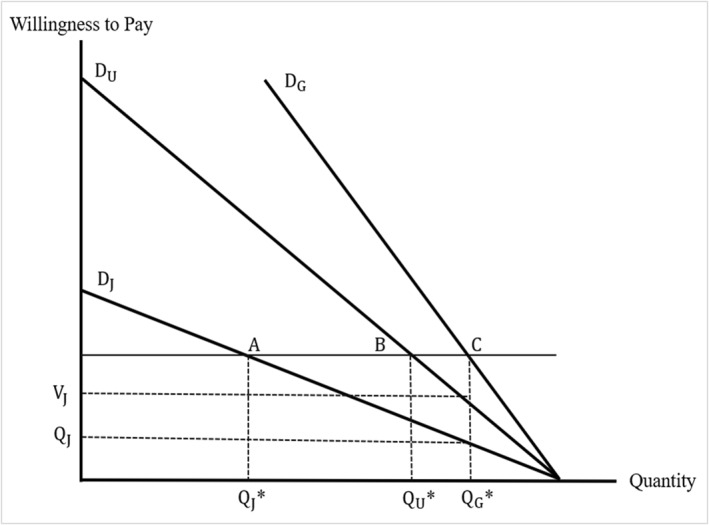
Optimal quantity of public good and willingness to pay.

Now assume that all citizens have identical valuations at *V*_*U*_, so that a country's demand curve at any Q for a population of *N*_*U*_ persons is then *N*_*U*_ *V*_*U*_ *(Q)*. This demand (or marginal valuation or willingness‐to‐pay) curve is shown as *D*_*U*_ in Figure 1. It represents the vertical sum of the marginal valuations of all residents of country *U*.

In addition, we can define a cost function representing the cost of bringing new pharmaceuticals to market as:

C=C(Q).

This cost includes both the probability of failure and the opportunity cost of capital during the period of pharmaceutical development. For simplicity, we assume that the marginal costs (MC) of production and distribution of pharmaceuticals is constant at zero. The resulting optimal quantity is then Q_U_* in Figure 1.

Now let there be another country *J* with the same individual valuations (*V*) as in country *U*, but with one third of the population. Its demand or marginal valuation curve is also derived as the sum of individual demand curves in Figure 1, as *D*_*J*_. The globally optimal quantity is then obtained by vertically summing the two country demand curves to yield *D*_*G*_, and the globally optimal quantity is QG*, which is larger than either isolated optimum.

Lindahl demonstrated that if the marginal contributions of each agent were set equal to their marginal valuation at the optimum, the resulting quantity would unanimously be preferred by all (Lindahl 1958). At the country level, the optimal total contributions would then be the marginal valuation times the optimal quantity. In that case, Country *J*'s contribution would be one third that of country *U*. Furthermore, under these assumptions, this outcome is reached if contributions per capita within each country are the same.

This simple model provides a definition of optimal support across countries for the public good of information embodied in new innovative pharmaceuticals. If there were demand responses from linking the contribution “tax” to the price of pharmaceuticals, then the optimal quantity might be slightly reduced along lines of the theory of optimal taxation (Johnson and Pauly 1969; Atkinson and Stern 1974). Furthermore, if other countries had higher or lower values of *V*, their contributions would vary with those values. In our assumed circumstances, where *V* is determined by GDP per capita alone, and also the elasticity of *V* with respect to GDP per capita is one, *V* (and the ideal contribution per capita) would be exactly proportional to GDP per capita.

Figure 1 also describes why this ideal outcome might not be achieved. Suppose country *U* moved first and set its contribution level at its individual optimum, *Q*_U_*. At that supply of new pharmaceuticals, the smaller country *J* would have a marginal valuation below marginal cost and would therefore not independently supply any of the associated public good. The country with the higher total valuation would pay more for the pharmaceutical than any smaller country. In the simple graphical analysis offered here, there is a corner solution, with the smaller country contributing nothing to the global public good, while the larger country bears the entire cost. As we note below, this outcome can be avoided through an agreement among them through which national contributions are determined.

## 5 A Positive Model of the Supply of Global Public Goods

The Olson‐Zeckhauser paper focused on the global public good of defense against aggression. It derived two important features of most alliances. The first is that “individuals” (here countries) acting independently will underprovide the public good. And the second feature is that “larger countries are likely to bear a disproportionate share of the burden” (1966, p. 267, 268). This theoretical result is commonly referred to as the “exploitation hypothesis.”

In the discussion below, we avoid discussing detailed political issues in individual countries. Instead, we presume that all measures taken depend entirely on a country's underlying economic conditions and citizen preferences. We envision states internationally as private actors, motivated by national self‐interest. This is a common approach employed in both the economic and political science literature on alliances and international cooperation (Kaul et al. 1999, 15).^1^ In this literature, there is the presumption that that each country makes optimal decisions for its own citizenry.

## 6 The Nash Non‐Cooperative Model

While the economic model developed here is similar to that offered originally by OZ, we employ the more formal approach of Bergstrom et al. (1986). We model the quantity of the relevant public good as the aggregate number of comparable new beneficial pharmaceuticals (comparable in the sense of having the same marginal health benefits per capita and the same R&D costs). Consider a world of *n* countries with each one potentially purchasing pharmaceuticals at prices above their marginal cost of production and distribution. In those circumstances, each country possibly contributes to the quasi‐rents that incentivize R&D efforts. The total number of new advanced pharmaceuticals, *Q*, is then supported by the aggregate contributions of *n* countries divided by the assumed uniform cost of the R&D sufficient to produce an additional pharmaceutical, indicated by *C*.

Each country is modeled as having a well‐behaved utility function for two goods reflecting both its own consumption of its private good, *x*_*i*_ and also the global aggregate of innovative new pharmaceuticals that results from all countries' R&D outlays. It maximizes the utility of its citizenry subject to a country specific budget constraint, yielding the demand or marginal benefit curves described above in Section II, Figure 1. There is no country subscript on the public good, Q, because all countries consume the same quantity of new, more efficacious pharmaceuticals. Each country then decides how much if any amount it will contribute toward the global public good based on its own demand or marginal valuation curve along with the cost of R&D.

Finally, let each country assume that its contribution will not influence the contributions of any other country. At the Nash noncooperative equilibrium, no country wishes to change its strategy, given the strategies of all other countries. Each country's contribution then represents its best response to the contributions of all others. Under these conditions, we can define a Nash equilibrium as the vector of contributions by all countries $\left(R_{i}^{*}\right),i=1,2\text{…}n$ that are independently utility maximizing.

In a Nash model with an interior equilibrium, more than one country contributes payment toward the global public good. However, a Nash corner equilibrium is also possible where one country optimally contributes such a large amount to producing the global public good while no other country willingly contributes anything. That result would be consistent with the arguments of both the CEA Reports and the Hooper/Henderson article: given the flow of new pharmaceuticals supported by US prices, no other country values another new pharmaceutical at greater than the pharmaceutical's own R&D cost.^2^

Another possible outcome is that some countries are at a corner solution and contribute nothing, while more than one country contributes something. A corner solution is likely if one country is much larger than all others. In the latter scenario, all smaller ROW countries pay just enough for their pharmaceuticals to cover the marginal cost of production and distribution. Note that in this model, whenever one country contributes more, each other country contributes less but instead spends more on its available private goods.

**FIGURE 2:**
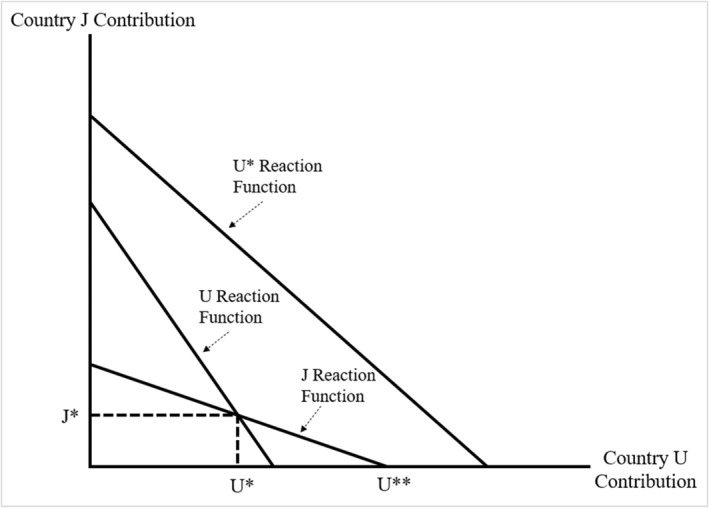
Reaction function and nash equilibrium.

Figure 2 further illustrates this process. It depicts reaction functions of the two countries: *U* and *J* in terms of an independent adjustment provision of the public good. If the reaction‐function lines cross, their intersection shows the division of outlays used to produce the public good. If, instead, the reaction function for country *U* is everywhere above the reaction function for *J* as is U* in Figure 2, there is a corner solution in which the large country *U* is the only supplier of the public good.

## 7 Other Factors Affecting Pricing

Other factors could potentially lead to deviations from simple Nash behavior. One is the presence of bilateral bargaining between ROW countries and pharmaceutical companies that generally occurs. In that case, each side has something to lose if an agreement is not reached. While companies would lose the prospective profits or quasi‐rents from lack of an agreement, ROW countries would lose their timely access to advanced new pharmaceuticals (Danzon et al. 2005; Pham et al. 2023).

Another factor is the widespread judgment that “value‐based pricing” should be the guiding principle for government price setting.^3^ Following that paradigm, some ROW countries may choose to reward successful high‐value innovations by paying higher prices that exceed marginal costs. While such prices will incentivize R&D efforts to some extent, we report below that they appear less consequential than those found in the United States. Both factors point to public good contributions higher than expected in a Nash equilibrium, at least for some countries.

## 8 How Pharmaceutical Prices Determine Contributions to the Global Public Good

To calculate national contributions to the global public good, we employ data on the prices paid for new branded pharmaceuticals from a study by the RAND Corporation.^4^ These data include price indices for 33 OECD countries in 2018 (Mulcahy et al. 2021).^5^ These indices can be stated as percentages of US prices, with the US index set at 100. For example, the price index for brand‐name originator pharmaceuticals for the UK is calculated as 43% or 0.43, which indicates the UK price index is 43% of the US index for branded pharmaceuticals. Coincidentally, the value reported for the total of all ROW countries is also 43% of the US index.

Next, we calculate the total sales revenue of branded pharmaceuticals from the RAND study by multiplying total sales by the proportion of sales involving branded pharmaceuticals. Returning to the UK example, we find total brand name sales of

$$S_{UK}=\left(\$23B\right)\;\left(0.71\right)=\$16.83B$$

At this point, we use a market‐based procedure to estimate marginal costs (MC) based on the data provided in the RAND report. Since firms will not accept prices below marginal costs, this approach is to use the price index of the lowest paying country, Turkey, as a rough estimate of marginal cost. This simple procedure suggests an MC value of 14.2% of the US price index. That estimate, however, may be too low; perhaps influenced by either a different basket of pharmaceuticals or lower distribution costs in this low‐income country. As a sensitivity test, we also employ a larger market‐based MC estimate of 24% of the US price index as described in the Appendix. This estimate is 9.8% points higher. Our basic findings, both descriptive and regression‐based, are robust to assumptions about the different values of MC.

By applying an MC value of 14.2% of the US price index, we can then derive each country's contribution from the proportion of the sales revenue each country provides that exceeds *MC* times total revenues from new branded pharmaceuticals:

$$\text{the}\;i^{\text{th}}\text{Countr}y^{\prime }s\;\text{Contribution}=\frac{\left(P_{i\;}‒\text{MC}\right)}{P_{i\;}}S_{i}$$

In this expression, $P_{i}$ is price index of innovative pharmaceuticals in country *i,* $S_{i}$ is the associated innovative pharmaceutical revenues, and *MC* is the estimated marginal cost assumed to be the same in all countries. Note that this measure is a country's *total* contribution to the public good, and not its contribution per capita.

Using the estimate of MC at 14.2% of the US price, each sample country's contribution to the global public good can be determined as:

$$\frac{\left[P_{i}-\left(P_{US\;}\right)\left(0.142\right)\right]}{P_{i}}\left({TS}_{i}\right)\left({BS}_{i}\right)$$

Where *TS*_*i*_ is total sales and *BS*_*i*_ is the proportion of sales that are branded pharmaceuticals.

An example is the UK contribution to the global public good:

$$\frac{0.43-0.142\;}{0.43}\left(\$23.7B\right)\left(0.71\right)=\$11.23B$$

In a parallel analysis for the US, we have:

$$\frac{\left[P_{US}-\left(P_{US\;}\right)\left(0.142\right)\right]}{P_{US}}S_{US}$$

which provides a US contribution of:

$$\frac{1.0-0.142}{1.0}\left(\$464.0\right)\left(0.82\right)=\$326.44B$$

The UK contribution is thus about 3.5% of the US total contribution, and about 17% of it in per capita terms.

As noted, the US contribution is $326.44B. In contrast, ROW contributions, measured here by the rest of the OECD, are:

$$\frac{0.43-0.142}{0.43}\left(\$331.2\right)\left(0.73\right)=\$164.28B$$

These calculations, therefore, indicate that the US contribution toward the global public good is approximately 66% of the total. This estimate is broadly consistent with prior estimates of between 64% and 78% (Goldman and Lakdawalla 2018), and also with estimates by Chen et al. (2023) using a different approach than employed here. See Table 1 for the public good contributions calculated for individual countries, and also the Appendix for further details.

**TABLE 1:** Calculated contributions and other data.

Country	Contribution, MC = 14.2% ($ Billions)	Cont per cap, MC = 14.2% ($)	GDP ($ trillions)	Population (millions)	GDP per capita ($ thousands)	BRAND‐name price index
Australia	4.52	181.5	1.28	24.9	51.56	38.00
Austria	2.63	295.47	0.49	8.89	55.40	50.75
Belgium	3.18	276.66	0.58	11.48	50.13	46.08
Canada	10.59	285.69	1.82	37.07	48.97	50.82
Chile	0.25	13.43	0.44	18.73	23.68	32.39
Czechia	1.31	122.51	0.42	10.67	39.82	42.60
Estonia	0.05	36.91	0.05	1.32	35.56	21.81
Finland	1.37	247.6	0.26	5.52	46.77	47.54
France	14.64	217.89	2.97	67.19	44.14	42.80
Germany	21.96	264.17	4.28	83.12	51.43	53.35
Greece	0.67	63.8	0.3	10.52	28.30	20.80
Hungary	1.13	116.47	0.31	9.71	31.78	42.20
Ireland	1.33	276.93	0.44	4.82	90.30	50.06
Italy	16.77	276.58	2.5	60.63	41.22	47.38
Japan	40.4	317.57	4.98	127.2	39.18	48.55
Latvia	0.11	56.97	0.06	1.93	30.29	30.78
Lithuania	0.17	60.14	0.1	2.8	35.04	27.03
Luxembourg	0.1	171.28	0.07	0.6	111.70	33.42
Mexico	1.61	12.76	2.47	126.19	19.58	40.63
Netherlands	1.68	98.19	0.94	17.06	55.09	39.57
New Zealand	0.51	108.31	0.2	4.74	41.28	43.83
Norway	1.31	246.04	0.34	5.34	64.11	42.39
Poland	2.34	61.61	1.19	37.92	31.50	41.91
Portugal	1.76	172	0.34	10.26	33.30	39.98
Rep. of Korea	3.42	66.82	2.1	51.17	41.08	28.01
Slovakia	0.56	103.36	0.17	5.45	31.67	34.81
Slovenia	0.33	160.81	0.08	2.08	38.51	37.37
Spain	13.03	279.13	1.9	46.69	40.67	50.21
Sweden	2.29	230.02	0.55	9.97	55.39	44.93
Switzerland	3.02	354.11	0.61	8.53	71.62	52.66
Turkey	0	0	2.21	82.34	26.86	14.20
United Kingdom	11.23	167.31	3.08	67.14	45.88	42.72
United States	326.44	998.01	20.37	327.1	62.27	100.00
Total world without US	164.28	170.77	37.53	961.98	39.01	43.00
Total world including US	490.72	380.67	57.90	1289.08	44.92	56.68

On a per capita basis as well, the US predominance is evident with contributions of $998 followed by Switzerland and Japan at $354 and $318 respectively. Other countries with substantial contributions include Canada, Spain, Austria, Belgium, and Germany. Overall, the other OECD countries make substantial contributions to the global public good of pharmaceutical innovation, representing about one third of total global support. The suggestion that other advanced countries are all paying near *MC*, and thus are entirely free‐riding, is not supported by these data. The free‐riding of these countries, while substantial, is not complete.

To be sure, national behavior close to pure free riding exists, with a number of countries making minimal contributions in total and per capita. See for example the values reported in Table 1 for Chile, Mexico, Greece, Estonia and Turkey. For these countries, their pricing practices approximate a corner solution in the OZ model.

## 9 The Determinants of Contributions: Empirical Estimates

### 9.1 Basic Approach and Variable Definitions

To explore the importance of aggregate demand on national support levels for this global public good,^6^ we estimate various single‐equation OLS models across the OECD countries included in the RAND report. Because of data limitations, the sample size is small at 32 countries. This is a cross‐sectional study, so it predominantly reflects long‐run equilibrium effects. As emphasized in the classic OZ paper and in a prior economic study of investment in pharmaceutical innovation (Acemoglu and Linn, 2004), our primary explanatory variable is the size of the economy as measured by its national GDP.^7^ A country's economic size determines the total value of the public good consumed by its citizenry, both because larger populations imply more people who benefit from a new pharmaceutical and also because higher GDP levels per capita are typically associated with higher values of improved health. Indeed, the demand for health has generally been reported to have an income elasticity of roughly 1.0 (Hemmitt and Robinson 2011; Viscusi and Masterman 2017).

In contrast to the US, no other OECD country has pursued a policy of directly promoting enhanced investment in pharmaceutical innovation as the US did in the Hatch Waxman Act. There is a prospect, however, that nations with large pharmaceutical industries will permit higher prices for other reasons. We therefore account for this possibility by sequentially adding indicator variables for countries with substantial pharmaceutical industries including the US, Japan, Germany and Switzerland. The version with only the US indicator is also a check on whether the results are driven by the US value.

### 9.2 Specifications

The baseline specification is a two‐variable linear regression equation:

$$C_{i\;}=\alpha +\beta_{1}{GDP}_{i}+\varepsilon_{i}$$

These equations are estimated in natural logs for the continuous variables so that the estimated coefficients represent elasticities. The dependent variable is the log of a country's total contribution to the global public good through prices that exceed MC. See Table 2 for descriptive statistics and correlations. Because the relevant size of a country is inherently multiplicative, separating the impact of population from GDP per capita is difficult; see the discussion and estimates in the Appendix.

**TABLE 2:** Descriptive statistics and correlations (*n* = 33).

	Mean	SD	Min	Median	Max
Descriptive statistics					
Contribution (MC = 14.2%), $ billions	14.9	56.6	0.0	1.7	326.4
Contribution per capita (MC = 14.2%), $	192.1	176.8	0.0	171.3	998.0
GDP, $ trillions	1.8	3.6	0.0	0.6	20.4
Population (millions)	39.1	62.5	0.6	10.7	327.1
GDP per capita, $ thousands	45.9	18.7	19.6	41.2	111.7
BRAND‐name price index	41.8	14.2	14.2	42.4	100.0

	Contribution, MC = 14.2% ($ Billions)	Cont per cap, MC = 14.2% ($)	GDP ($ trillions)	Population (millions)	GDP per capita ($ thousands)	BRAND‐name price index
Correlations						
Contribution (MC = 14.2%)	1	0.853	0.970	0.876	0.152	0.771
Cont per capita (MC = 14.2%)	0.853	1	0.818	0.688	0.437	0.920
GDP	0.970	0.818	1	0.958	0.098	0.753
Population	0.876	0.688	0.958	1	−0.033	0.654
GDP per capita	0.152	0.437	0.098	−0.033	1	0.352
BRAND‐name price index	0.771	0.920	0.753	0.654	0.352	1

### 9.3 Empirical Findings: Country Size

**FIGURE 3:**
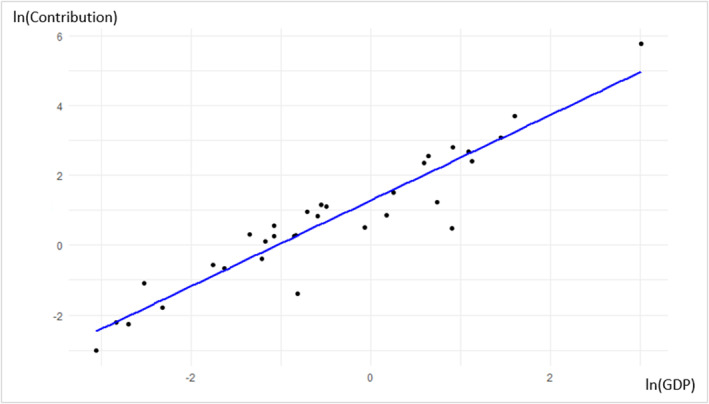
Scatter plot, country size and contribution. Mc = 14.2% of US prices.

A country's GDP explains a large share of its contribution to the global public good. See Table 3 and the scatter plots in Figure 3. In that plot, the upper right observation is the US. As one can see, the data line up nicely when linear in logs. Because innovative pharmaceuticals are used in all OECD countries, it is apparent that variation in national contributions depends overwhelmingly on GDP levels.

**TABLE 3:** Explaining contribution by country MC = 14.2% of US price index.

Dependent variable:	Log of total contribution (MC = 14.2%)				
Model:	(1)	(2)	(3)	(4)	(5)
Variables					
Constant	1.28***	1.22***	1.19***	1.17***	1.15***
	(0.132)	(0.141)	(0.151)	(0.170)	(0.177)
Log of GDP ($ trillions)	1.23***	1.17***	1.14***	1.13***	1.13***
	(0.078)	(0.085)	(0.093)	(0.107)	(0.109)
1 (US)		1.03**	1.16**	1.21*	1.24*
		(0.361)	(0.397)	(0.459)	(0.471)
1 (Japan)			0.678*	0.715*	0.739*
			(0.274)	(0.317)	(0.326)
1 (Germany)				0.279	0.303
				(0.301)	(0.310)
1 (Switzerland)					0.513**
					(0.144)
Fit statistics					
Observations	32	32	32	32	32
R^2^	0.885	0.893	0.896	0.897	0.899
Adjusted R^2^	0.881	0.885	0.885	0.882	0.880
Dependent variable mean	0.70	0.70	0.70	0.70	0.70

*Note:* Heteroskedasticity‐robust standard‐errors in parentheses. Signif. Codes: ***: 0.01, **: 0.05, *: 0.1.

A regression that includes ln GDP as the sole explanatory variable explains 89% of the variation in the log of contributions across countries and is highly significant at better than the 1% level, with a t‐statistic of 16; see Table 3, Column 1. Furthermore, the size effect, as reflected by its GDP, is quantitatively large with an estimated point elasticity of 1.23. The arc elasticity indicating the effect of doubling the GDP variable is even higher at 1.45 due to the non‐linear property of a logarithmic equation.^8^ This equation therefore projects that, on average, doubling GDP leads to increased contributions to the Global Public Good by 145%. These findings appear despite the reference pricing and *trans*‐shipments that suppress price variation among OECD countries (Danzon 2018; Salant 2023; Maini and Passamoli 2023).

These estimates are also consistent with the “exploitation hypothesis,” discussed earlier, which holds that there is a tendency for the “small” to exploit the “large.” In contrast, if public good contributions were made proportionately to a country's GDP, the regression coefficient would approach unity. As reported in Table 3, Column (1), the estimated coefficient is greater than one with a t‐statistic for the difference from one of 2.95 (0.23/0.078) that is significant at the 1.0% level in a two‐tailed test. An “exploitation” result would offer an explanation for the higher US pharmaceutical prices as compared with those in other advanced countries.

The simple specification is tied directly to Olson's (1965) theoretical analysis where group (or country) size is an important factor for the actions taken. Indeed, country size as measured by its GDP explains 89% of the variation in country‐level contribution. Explaining 89% of the variation leaves little room for other variables to make much of a difference. Furthermore, this result is robust to different (larger) estimate of marginal costs, to adding dummies for countries with large pharmaceutical industries, and to adding controls for other aspects of the health care system and pharmaceutical regulation.

To separate a possible US effect from a more generalized size effect, we introduce a US indicator variable, as reported in Table 3, Column 2. As indicated in that table, both the US variable and the generalized size effect remain highly statistically and economically significant. While the size coefficient is slightly lower than before at 1.17 rather than 1.23, it remains significant, with a t‐statistic of 14. Despite its large size and large contribution, the US observation does not drive the underlying size effect.

Note that the data are again consistent with the “exploitation” hypothesis, although the estimates are less precise than before. The comparable test statistic based on Table 3, Column 2 is now 2.0 (0.17/0.085) which is statistically significant at the 10.0% level and nearly statistically significant at the 5% level in a two‐tailed test. Even after accounting for the distinct US effect, the data are consistent with the “exploitation hypothesis”.

We next add indicator variables for three other countries with notable pharmaceutical industries: Japan, Germany and Switzerland. These variables are all positive and, except for Germany, and statistically significant at the 5% level. However, the precision of the estimates declines with the larger set of indicators. The adjusted R^2^ is highest with only the US indicator variable included in the equations, remains the same with the addition of Japan, but then declines when other country indicator variables are added.

Pertile et al. (2018) take a similar conceptual approach and obtain generally supportive findings. One difference is that they emphasize dynamics and the estimation of Nash reaction functions. On the empirical side, their study is limited to 70 cancer pharmaceuticals, while our study uses the universe of branded pharmaceuticals. Their study has a smaller sample size of 25. Their study examines prices, while we calculate and analyze each country's contributions to the global public good.^9^

Interestingly, an additional study that focused on a different global public good, government and foundation funded basic medical research, employed a similar theoretical framework and reaches similar conclusions. As a percentage of GDP, the US contributions to basic medical research are triple the contributions of all other OECD countries except for the UK. The authors provide a similar theoretical explanation as that developed here (Kyle et al. 2017, 191, 192).

## 10 Conclusions

Viewing pharmaceutical profits through the lens of the global public good of therapeutic information created by industry R&D leads to valuable insights. Following the classic work of OZ, this analysis projects a global deficit in pharmaceutical innovation; and also that large countries bear the largest burden. This analysis thereby explains why the US pays so much more for patented pharmaceuticals than do other rich countries.

In our analysis, we define a Lindahl equilibrium that describes worldwide optimality but also explain why its achievement is unlikely. Nationally self‐interested behavior causes support for the public good to fall short of optimality. There is no global authority to enforce Lindahl prices even if they could be determined. However, when some ROW countries appear to set their buying prices, even partially, on therapeutic improvements rather than on price‐minimization, and when pharmaceutical companies can negotiate prices exceeding marginal costs, a country's contributions to the global public good expands beyond marginal cost.

If one were to assume that the level of short‐term profit flows for new branded pharmaceuticals in the US were generally closer to the ideal, then per capita contributions of ROW countries with the similar GDP per capita as the United States should be roughly similar—and that contributions should be adjusted as GDP per capita varies. It thereby seems evident that rich countries now making small contributions, such as the Netherlands and Norway, should ideally contribute more to the discovery of new pharmaceuticals.

Future policy discussions should emphasize the global nature of the public good and the possible gains resulting from international cooperation. There are mutually beneficial opportunities for increasing global support for this global public good. We agree with the Goldman and Lakdawalla argument that “if other wealthy countries shouldered more of the burden for medical innovation, both American, European and Japanese patients would benefit. More can be done through trade deals” (2018, 4, 5).^10^ Current outcomes could be improved through international agreements.

Efforts to create multi‐country agreements leading to greater revenues received from ROW countries while keeping US‐generated revenues at current levels would represent a strong step in that direction. That approach would resemble international trade agreements, environmental agreements (e.g, The Montreal Protocol on ozone depletion) and even defensive alliances. International cooperation can convert the current non‐cooperative game to one more likely to promote enhanced global public health.

## Conflicts of Interest

The authors declare no conflicts of interest.

## Data Availability

Data sharing not applicable to this article as no datasets were generated or analysed during the current study.

## Endnotes

^1^
Similar presumptions underlie works by Egan and Philipson (2013) and the US CEA (2018).

^2^
For a new paper examining the conditions for a single‐contributor Nash equilibrium, see Bergstrom (2025).

^3^
See the arguments for this approach advanced in (Neumann et al. 2021).

^4^
The category excludes “Brand‐Name Non‐Originators.” That category is small and US prices for these pharmaceuticals are much closer to ROW prices. Therefore, it contributes little to the global public good.

^5^
RAND has recently done the analysis for 2022, using a slightly different methodology. There is a strong, positive correlation between the years, *r* = 0.86.

^6^
The importance of group (country) size was original explored by Olson,1971.

^7^
For more on measures of country size for global public good problems, including the use of total GDP, see (Shresthaand Feehan 2002/2003).

^8^
Doubling GDP raises lnGDP by 0.7, so that lnCont increases by (0.7) (1.23) = 0.86. Taking the antilog, this raises Cont by 1.45 or 145%.

^9^
This paper was pointed out to us by a referee.

^10^
Kyle et al. (2017, 194) make a similar argument for greater international coordination in the context of a different global public good, direct support for basic medical research, discussed above.

## Calculating Country‐Level Contributions to the Global Public Good: Details

### Expressing Relative Prices in the Appropriate Form

The relative prices from the RAND Report are expressed in different form than employed here. They are expressed as US prices relative to individual OECD country prices, provided in percentage terms:

$$US\;Price\;Premium\;for\;the\;i^{th}Country=\frac{P_{US}}{P_{i}}\left(100\right)$$

For our purposes, we transform those price indices into proportions of the corresponding US prices. To this end, we invert the published indices for the innovative pharmaceuticals, and multiply by 100 so that the price index for the *ith* country is:

$$P_{i}=\frac{1}{US\;Price\;Premium\;for\;the\;i^{th}Country}\left(100\right)$$

### Sensitivity Analysis

In our first test, we estimate regression equations using the alternate higher estimate of MC at 24% of US prices. The results are very similar but with slightly larger size effects. As indicated below, the basic results are quite robust to different MC estimates. We also estimated the regression equations in linear rather than logarithmic form for the continuous variable. The results obtained are again similar to those reported above.

We also experimented with two types of specifications focusing directly on per capita GDP. First, we estimated a per capita version by dividing our data by population. This gave results suggesting very disproportionate per capita contribution with respect to the per capita income aspect of size, again consistent with the “exploitation hypothesis”. The term involving population was imprecisely estimated and the overall fit was relatively poor.

We next relaxed our assumption that the variables chosen to affect contribution to the global public good were fully endogenous with respect to the country's intended contribution. For this purpose, we included plausible control variables for the out‐of‐pocket cost of health care along with the threshold value of cost‐effectiveness required by national authorities or insurance companies to accept pharmaceuticals for payment. Neither variable is significant at even the 10% level, although the cost‐effectiveness threshold parameter was nearly statistically significant in one specification. Most important, the size effect was minimally affected by the presence of these variables. The basic results thus appear highly robust to alternate specifications.

Note that simply regressing total contribution on GDP per capita and population is surely mis‐specified. It implies that the effect of increasing population is independent of income per capita and vice versa. The way to clarify the issues is to estimate an equation for total contribution as a function of size (GDP) and per capita income, which is the second approach we pursued. Our result was that per capita income added little to the regression equation and had almost no effect on the key elasticity of contributions with respect to size of the country.

### Alternative Estimates of Marginal Cost

As discussed above, our primary approach is to use the price index of the lowest paying country, Turkey, as an estimate of marginal cost. This simple procedure suggests a MC value of 14.2% of the US price index.^11^ That estimate, however, may be too low.

As a robustness test, we also use another market‐based estimate in order to compare our main results with those based on an alternative estimate taken from the economic literature and summarized in a recent CEA Report. The estimate is based on studies that calculate the average US price declines resulting from significant generic entry (Grabowski et al. 2002; Berndt et al. nov. 2011). The fundamental concept employed here is that prices approach marginal costs when substantial generic entry occurs. The CEA reviewed these studies and reported that the average estimated price decline following large‐scale generic entry for the studies reviewed is 84%, implying average marginal costs of 16% of the earlier *list* price (US Council of Economic Advisors 2018, 24). This estimate, however, must be adjusted for the average US rebate of 33% for branded pharmaceuticals (Mulcahy et al. 2021, 25). In that case, we estimate an average *MC* value of 24% of net US prices, or 0.24.^12^

Resulting estimates of country‐by‐country contributions for the two alternative estimates of MC can be seen in Tables A1 and A2. For more convenient comparison, these tables repeat the relevant results from the body of the paper.

**TABLE A1:** Descriptive statistics and correlations, alternative marginal costs.

	Mean	SD	Min	Median	Max
Descriptive statistics					
Contribution (MC = 24%), $ billions	12.1	50.1	−2.4	1.0	289.2
Contribution (MC = 14.2%), $ billions	14.9	56.6	0.0	1.7	326.4
Contribution per capita (MC = 24%), $	130.6	158.8	−31.0	106.6	884.0
Contribution per capita (MC = 14.2%), $	192.1	176.8	0.0	171.3	998.0
GDP, $ trillions	1.8	3.6	0.0	0.6	20.4
Population (millions)	39.1	62.5	0.6	10.7	327.1
GDP per capita, $ thousands	45.9	18.7	19.6	41.2	111.7
BRAND‐name price index	41.8	14.2	14.2	42.4	100.0

	Contribution, MC = 24% ($ Billions)	Contribution, MC = 14.2% ($ Billions)	Cont per cap, MC = 24% ($)	Cont per cap, MC = 14.2% ($)	GDP ($ trillions)	Population (millions)	GDP per capita ($ thousands)	BRAND‐name price index
Correlations								
Contribution (MC = 24%)	1	1	0.881	0.850	0.964	0.867	0.156	0.770
Contribution (MC = 14.2%)	1	1	0.883	0.853	0.970	0.876	0.152	0.771
Cont per capita (MC = 24%)	0.881	0.883	1	0.994	0.850	0.728	0.392	0.940
Cont per capita (MC = 14.2%)	0.850	0.853	0.994	1	0.818	0.688	0.437	0.920
GDP	0.964	0.970	0.850	0.818	1	0.958	0.098	0.753
Population	0.867	0.876	0.728	0.688	0.958	1	−0.033	0.654
GDP per capita	0.156	0.152	0.392	0.437	0.098	−0.033	1	0.352
BRAND‐name price index	0.770	0.771	0.940	0.920	0.753	0.654	0.352	1

**TABLE A2:** Calculated contributions and other data alternative marginal costs.

Country	Contribution, MC = 24% ($ Billions)	Contribution, MC = 14.2% ($ Billions)	Cont per cap, MC = 24% ($)	Cont per cap, MC = 14.2% ($)	GDP ($ trillions)	Population (millions)	GDP per capita ($ thousands)	BRAND‐name price index
Australia	2.66	4.52	106.77	181.5	1.28	24.9	51.56	38.00
Austria	1.92	2.63	216.26	295.47	0.49	8.89	55.40	50.75
Belgium	2.2	3.18	191.62	276.66	0.58	11.48	50.13	46.08
Canada	7.76	10.59	209.24	285.69	1.82	37.07	48.97	50.82
Chile	0.12	0.25	6.19	13.43	0.44	18.73	23.68	32.39
Czechia	0.86	1.31	80.25	122.51	0.42	10.67	39.82	42.60
Estonia	−0.01	0.05	−10.64	36.91	0.05	1.32	35.56	21.81
Finland	0.97	1.37	174.82	247.6	0.26	5.52	46.77	47.54
France	9.62	14.64	143.25	217.89	2.97	67.19	44.14	42.80
Germany	16.46	21.96	198.06	264.17	4.28	83.12	51.43	53.35
Greece	−0.33	0.67	−30.99	63.8	0.3	10.52	28.30	20.80
Hungary	0.74	1.13	75.72	116.47	0.31	9.71	31.78	42.20
Ireland	0.97	1.33	201.26	276.93	0.44	4.82	90.30	50.06
Italy	11.82	16.77	194.91	276.58	2.5	60.63	41.22	47.38
Japan	28.87	40.4	226.99	317.57	4.98	127.2	39.18	48.55
Latvia	0.04	0.11	23.3	56.97	0.06	1.93	30.29	30.78
Lithuania	0.04	0.17	14.21	60.14	0.1	2.8	35.04	27.03
Luxembourg	0.05	0.1	83.94	171.28	0.07	0.6	111.70	33.42
Mexico	1.01	1.61	8.03	12.76	2.47	126.19	19.58	40.63
Netherlands	1.03	1.68	60.26	98.19	0.94	17.06	55.09	39.57
New Zealand	0.34	0.51	72.5	108.31	0.2	4.74	41.28	43.83
Norway	0.86	1.31	160.53	246.04	0.34	5.34	64.11	42.39
Poland	1.51	2.34	39.82	61.61	1.19	37.92	31.50	41.91
Portugal	1.09	1.76	106.62	172	0.34	10.26	33.30	39.98
Rep. of Korea	0.99	3.42	19.41	66.82	2.1	51.17	41.08	28.01
Slovakia	0.3	0.56	54.23	103.36	0.17	5.45	31.67	34.81
Slovenia	0.19	0.33	92.8	160.81	0.08	2.08	38.51	37.37
Spain	9.49	13.03	203.17	279.13	1.9	46.69	40.67	50.21
Sweden	1.56	2.29	156.67	230.02	0.55	9.97	55.39	44.93
Switzerland	2.25	3.02	263.89	354.11	0.61	8.53	71.62	52.66
Turkey	−2.37	0	−28.76	0	2.21	82.34	26.86	14.20
United Kingdom	7.37	11.23	109.83	167.31	3.08	67.14	45.88	42.72
United States	289.16	326.44	884.04	998.01	20.37	327.1	62.27	100.00
Total world without US	110.39	164.28	114.75	170.77	37.53	961.98	39.01	43.00
Total world including US	399.55	490.71	309.95	380.67	57.9	1289.08	44.92	56.68

The relative positions of the countries are similar, although the quantitative estimates are somewhat different. Naturally, all calculated contributions are lower for MC = 24% than for MC = 14.2%. Turning to the regressions explaining contributions, see Tables A3 and A4. Table A3 reprints the results for MC = 14.2%, while Table A4 shows the results for MC = 24%. The basic results are similar, showing the they are robust to quite different estimates of marginal cost.

**TABLE A3:** Explaining contribution by country. MC = 14.2% of US price index (reprinted from Table 3 in the paper for convenient comparisons).

Dependent variable:	Log of total contribution (MC = 14.2%)				
Model:	(1)	(2)	(3)	(4)	(5)
Variables					
Constant	1.28***	1.22***	1.19***	1.17***	1.15***
	(0.132)	(0.141)	(0.151)	(0.170)	(0.177)
Log of GDP ($ trillions)	1.23***	1.17***	1.14***	1.13***	1.13***
	(0.078)	(0.085)	(0.093)	(0.107)	(0.109)
1 (US)		1.03**	1.16**	1.21*	1.24*
		(0.361)	(0.397)	(0.459)	(0.471)
1 (Japan)			0.678*	0.715*	0.739*
			(0.274)	(0.317)	(0.326)
1 (Germany)				0.279	0.303
				(0.301)	(0.310)
1 (Switzerland)					0.513**
					(0.144)
Fit statistics					
Observations	32	32	32	32	32
R^2^	0.885	0.893	0.896	0.897	0.899
Adjusted R^2^	0.881	0.885	0.885	0.882	0.880
Dependent variable mean	0.70	0.70	0.70	0.70	0.70

*Note:* Heteroskedasticity‐robust standard‐errors in parentheses. Signif. Codes: ***: 0.01, **: 0.05, *: 0.1.

**TABLE A4:** Explaining contribution by country. MC = 24% of US price index.

Dependent variable:	Log of total contribution (MC = 24%)				
Model:	(1)	(2)	(3)	(4)	(5)
Variables					
Constant	0.832***	0.764***	0.722***	0.695**	0.667**
	(0.163)	(0.173)	(0.187)	(0.209)	(0.217)
Log of GDP ($ trillions)	1.29***	1.21***	1.18***	1.16***	1.16***
	(0.104)	(0.115)	(0.128)	(0.146)	(0.148)
1 (US)		1.24*	1.39*	1.48*	1.51*
		(0.458)	(0.512)	(0.592)	(0.605)
1 (Japan)			0.746*	0.806	0.838
			(0.345)	(0.399)	(0.409)
1 (Germany)				0.422	0.453
				(0.379)	(0.389)
1 (Switzerland)					0.714***
					(0.181)
Fit statistics					
Observations	30	30	30	30	30
R^2^	0.834	0.844	0.848	0.850	0.854
Adjusted R^2^	0.828	0.833	0.831	0.826	0.824
Dependent variable mean	0.37	0.37	0.37	0.37	0.37

*Note:* Heteroskedasticity‐robust standard‐errors in parentheses. Signif. Codes: ***: 0.01, **: 0.05, *: 0.1.

### Alternative Specifications

#### Per Capita Basis

As a further check, we estimated regression equations on a per capita basis. The theoretically correct way to do this is to divide our original regression by population and then add a constant to avoid forcing the regression line through the origin. This procedure gives:

$$\frac{C_{i}}{Pop}=\alpha +\beta_{1}\frac{1}{{Pop}_{i}}+\beta_{2}\frac{GDP}{Pop}+\epsilon_{i}$$

Estimating that regression equation for both estimates of MC gives the regressions in Table A5.

**TABLE A5:** Regressions on a per capita basis.

Dependent variable:	Log of total contribution per capita in thousands, $ (MC = 24%)	Log of total contribution per capita in thousands, $ (MC = 14.2%)
Variables		
Constant	−19.66*** (5.131)	−14.38** (5.146)
1/(log of population in millions)	0.395 (0.397)	−0.012 (0.268)
Log of GDP per capita, $	2.246***	1.810***
	(0.475)	(0.481)
Fit statistics		
Observations	30	32
R	0.460	0.503
Adjusted R	0.420	0.468
Dependent variable mean	4.52	4.95

*Note:* Signif. Codes: ***: 0.01, **: 0.05, *: 0.1.

As one can see, the explanatory power of the per capita regression is less than in the main specification in terms of total GDP and total contribution. This result is not surprising since the dependent variable, per capita contribution, is different and does not vary nearly as much as does total contributions. Further, the effect of size is now more complex, involving two terms, one of which, the effect of 1/Pop, is imprecisely estimated. If we ignore that term, the elasticity of contributions with respect to per capita income is implausibly large, with an elasticity of 1.81 for the MC = 14.2% of US prices regression and 2.246 for the MC = 24% of US prices regression.

Since the coefficient on 1/Pop is so loosely estimated, we also estimated the regression equations excluding 1/Pop. The coefficient on GDP per capital was very similar for MC = 14.2% and smaller (but still large) for MC = 24%. The adjusted R^2^ is actually higher for the MC = 14.2% regression when excluding the 1/Pop. See Table A6. Taken literally, these per capita regressions indicate that larger economies, in terms of per capita income, contribute much more, whether population is held constant or not. This is consistent with the “small exploits the great” theory.

**TABLE A6:** Alternative regressions on a per capita basis.

Dependent variable:	Log of total contribution per capita in thousands, $ (MC = 24%)	Log of total contribution per capita in thousands, $ (MC = 14.2%)
Variables		
Constant	−16.69* (6.624)	−14.49* (5.318)
Log of GDP per capita, $	1.982**	1.820**
	(0.621)	(0.499)
Fit statistics		
Observations	30	32
R^2^	0.429	0.502
Adjusted R^2^	0.408	0.486
Dependent variable mean	4.52	4.95

#### Adding Population and GDP Cer Capita

As another check, we sought to add Pop and GDP per capita to our main regression. We were not able to add Pop, because ln (Pop) was so colinear with the other two variables that the software would not run. So, we just added ln (GDP per capita), giving us the following estimating equation:

$$C_{i\;}=\alpha +\beta_{1}{GDP}_{i}+\beta_{2}\frac{{GDP}_{i}}{{Pop}_{i}}\;\varepsilon_{i}$$

This equation provides a test for whether GDP/Pop adds explanatory power over and above the main specification using the size‐of‐the‐economy variable. See Table A7.

**TABLE A7:** Regressions adding GDP per capita to main specification.

Dependent variable:	Log of total contribution in billions, $ (MC = 24%)	Log of total contribution in billions, $ (MC = 14.2%)
Variables		
Constant	−9.641 (5.527)	−6.936 (4.823)
Log of GDP ($ trillions)	1.287*** (0.102)	1.216*** (0.078)
Log of GDP per capita, $	0.979 (0.513)	0.769 (0.448)
Fit statistics		
Observations	30	32
R^2^	0.866	0.907
Adjusted R^2^	0.856	0.900
Dependent variable mean	4.52	4.95

As one can see, GDP per capita adds essentially nothing to the explanatory power of size of the economy (total GDP). It is not significant and hardly changes the main estimates of the elasticity of contribution with respect to size.

In sum, the specification using total GDP and total contribution is more appropriate in theory and has better explanatory power than one which divides GDP into two parts, population and GDP/POP). Dividing GDP into two parts and entering population and GDP/POP linearly is especially problematic. Both theory and empirical evidence show that the influence of total GDP is not additively separable into these two components.
